# Effects of horizontal versus vertical switching of disease-modifying treatment after platform drugs on disease activity in patients with relapsing–remitting multiple sclerosis in Austria

**DOI:** 10.1007/s00415-023-11644-y

**Published:** 2023-03-02

**Authors:** Michael Guger, Christian Enzinger, Fritz Leutmezer, Franziska Di Pauli, Jörg Kraus, Stefan Kalcher, Erich Kvas, Thomas Berger, the Austrian MS Treatment Registry (AMSTR)

**Affiliations:** 1 Department of Neurology, Pyhrn-Eisenwurzen Hospital Steyr, Sierninger Straße 170, 4400 Steyr, Austria; 2 Medical Faculty, Johannes Kepler University Linz, Linz, Austria; 3 Department of Neurology, Medical University of Graz, Graz, Austria; 4 Department of Neurology, Medical University of Vienna, Vienna, Austria; 5 Comprehensive Center for Clinical Neurosciences and Mental Health, Medical University of Vienna, Vienna, Austria; 6 Clinical Department of Neurology, Medical University of Innsbruck, Innsbruck, Austria; 7 Department of Laboratory Medicine, Paracelsus Medical University and Salzburger Landeskliniken, Salzburg, Austria; 8 Department of Neurology, Medical Faculty, Heinrich-Heine-University, Düsseldorf, Germany; 9 Hermesoft, Data Management, Graz, Austria; 10 Hermesoft, Statistics, Graz, Austria

**Keywords:** Multiple sclerosis, Escalation, Horizontal, Vertical, Switch

## Abstract

**Objectives:**

To compare in a nationwide observational cohort the effectiveness, frequency and reasons for treatment interruption of dimethylfumarate (DMF) and teriflunomide (TERI) (horizontal switchers) versus alemtuzumab (AZM), cladribine (CLAD), fingolimod (FTY), natalizumab (NTZ), ocrelizumab (OCR) and ozanimod (OZA) (vertical switchers) in patients with relapsing–remitting multiple sclerosis (pwRRMS) and prior interferon beta (IFN-beta) or glatiramer-acetate (GLAT) treatment.

**Materials and methods:**

The “horizontal switch cohort” included 669 and the “vertical switch cohort” 800 RRMS patients. We used propensity scores for inverse probability weighting in generalized linear (GLM) and Cox proportional hazards models to correct for bias in this non-randomized registry study.

**Results:**

Estimated mean annualized relapse rates (ARR) were 0.39 for horizontal and 0.17 for vertical switchers. The incidence rate ratio (IRR) in the GLM model showed an increased relapse probability of 86% for horizontal versus vertical switchers (IRR = 1.86; 95% CI 1.38–2.50; *p* < 0.001). Analyzing the time to the first relapse after treatment switch by Cox regression, a hazard ratio of 1.58 (95% CI 1.24–2.02; *p* < 0.001) indicated an increased risk of 58% for horizontal switchers. The hazard ratios for treatment interruption comparing horizontal versus vertical switchers were 1.78 (95% CI 1.46–2.18; *p* < 0.001).

**Conclusions:**

Horizontal switching after a platform therapy resulted in a higher relapse and interrupt probability and was associated with a trend towards less EDSS improvement comparing to vertical switching in Austrian RRMS patients.

## Introduction

In comparison to placebo groups, dimethylfumarate (DMF) reduced the annualized relapse rate (ARR) in patients with multiple sclerosis (MS) by 44–53% [1, 2] and teriflunomide (TERI) by 32–36% [3, 4]. Both have, therefore, been approved for treatment of MS and are used for a mild to moderate disease course [5]. In respective trials, fingolimod (FTY) reduced the ARR by 48–54% [6, 7], natalizumab (NTZ) by 68% [8] and cladribine CLAD) by 58% [9, 10]. In contrast, alemtuzumab (AZM), ocrelizumab (OCR) and ozanimod (OZA) revealed superior efficacy versus a moderately effective comparator [11–15]. These MS drugs are, therefore, indicated for an active or highly active disease course [5].

Most clinicians wish to resort to an evidence-based decision in the case of treatment switches. However, studies comparing the clinical effectiveness between a horizontal and vertical switch after a platform therapy [interferon beta (IFN-beta) or glatiramer-acetate (GLAT)] [16] provided conflicting results [17–34]. These discrepancies ask for further investigations, especially based on real-life data.

We here, thus, sought to compare the effectiveness, frequency and reasons for treatment interruption in patients with prior IFN-ß or GLAT treatment switching to either DMF and TERI (horizontal switchers) or AZM, CLAD, FTY, NTZ, OCR and OZA (vertical switchers) in a nationwide observational cohort using prospectively collected data from a real-life setting.

## Materials and methods

### Data collection

The Austrian MS Treatment Registry (AMSTR) [17, 18], established in 2006 to maintain quality control and comply with reimbursement regulations of the Austrian sick funds, allows to obtain clinical data, assess indications, clinical profiles of treated patients and to monitor safety in real life. The AMSTR is part of the dense network of MS centers in Austria, which is constituted by MS clinics from neurological departments and some dedicated neurological practices that have been assigned this status by the Austrian Society of Neurology based on defined quality criteria. In addition, prescriptions of disease-modifying therapies (DMTs) for MS are exclusively reserved for MS centers. Thus, prescriptions and treatment documentations are evenly distributed across Austria. The AMSTR is compliant with Austrian laws on bioethics and it was also approved by the ethical committee of the Medical University of Vienna (EC number 2096/2013).

The AMSTR documents anonymous baseline data, including the date of clinical onset of MS and disease duration, relapses in the prior 12 months, EDSS, magnetic resonance imaging (MRI) activity and previous DMT. Follow-up data (relapses, EDSS, adverse events [AEs], change or discontinuation of treatment) are required to be documented every 3–6 months. Because of the structure and requirements of the AMSTR, MRI results were only available at baseline before start of treatment, but unfortunately not during follow-up. Each relapse has to be confirmed by a neurologist at the MS center and documented in the AMSTR. Documentation also requires date of relapse onset, EDSS and use/dose of i.v. methylprednisolone treatment. Besides the fact that applying the AMSTR is mandatory for reimbursement, external and independent data monitoring to improve data acquisition, input and management in terms of completeness and plausibility of documented data constitutes a special quality-related feature of the AMSTR.

We identified two cohorts of patients with relapsing–remitting multiple sclerosis (RRMS) from the AMSTR since 2006, who have either switched from IFN-beta and/or GLAT to DMF and TERI (‘horizontal switchers’) or to AZM, CLAD, FTY, NTZ, OCR and OZA (‘vertical switchers’). The horizontal switchers correspond to lateral switchers and the vertical switchers to escalation treatment as cited in the literature [33]. Patients had to remain on the switched treatment for at least three months due to disease activity, patients preference and side effects. The horizontal switch cohort included 669 and the vertical switch cohort 800 RRMS patients.

### Outcome measures

The primary outcome measure was the annualized relapse rate (ARR) under treatment during this period. Relapses were defined as new or worsening neurological symptoms lasting for at least 24 h in the absence of fever. Further outcome measures were time to first relapse and sustained disability progression or regression. Sustained disability progression or regression was defined as an increase or decrease from baseline of at least 1.0 point in the EDSS (or at least 0.5 points for patients with a baseline EDSS score greater than 5.5) that persisted for at least 12 or 24 weeks.

For analyses of treatment interruption, we defined two causes, namely (a) stopping treatment as permanent treatment interruption in the AMSTR and (b) switching treatment as treatment interruption and starting with a new medication in the AMSTR.

### Statistical methods

All effects estimated in comparing treatment groups were average treatment effects (ATE). To control the bias for non-randomized assignment to the treatment groups, we used normalized inverse probability weighting (IPW). Propensity scores for treatment in the horizontal and vertical switchers were estimated with a logistic regression model with the baseline parameters age, duration of disease, number of relapses 12 months prior to baseline, EDSS, presence of at least 9 cerebral MRI T2 lesions and at least one contrast-enhancing brain MRI lesion, and previous therapy as independent variables. These variables were included in the model because of their clinical meaning, independent from their significance as a predictor in the model. Therefore, we tried to overcome the problems of being misled by false-positive predictors in a multiple testing situation as well as of missing relevant variables by abandoning them in a beta failure decision. The study cohort was truncated for two patients producing weights higher than ten. Treatment groups were well balanced for all variables after weighting. The vertical switcher group produced higher propensity scores for their treatment on average, leading to a right tail of the propensity score distribution closer to one and a left tail shorter against zero compared to the propensity score distribution of the horizontal switcher group. This fact produced higher weights on the left and right sides of the propensity score distribution for the horizontal switcher group, leading to a higher sample size for the horizontal group after weighting.

A generalized linear model (GLM) with relapse count as Poisson distributed-dependent variable and log-transformed observation time in years as offset variable was used to estimate the treatment effect on the ARR in the observation period.

Augmented inverse probability weighting was used to analyze the change of EDSS from baseline to the last visit in the observation period, with the difference between last visit and baseline (negative as improvement, positive as worsening) for each patient as dependent variable. Potential means for these changes were estimated for each treatment from the model.

We used Cox proportional hazards models for analyzing EDSS progression and regression confirmed after 12 and 24 weeks, and the relapse hazard in the observation period.

All models included treatment as categorical factor and normalized inverse propensity scores as weights regarding the survey character of the study. All variables used for propensity scoring were also used in the outcome models as independent variables to obtain adjusted treatment effects. We applied this double robust approach, because the ATE estimator remains consistent if at least one of the two, the propensity score model or the outcome regression, is specified properly. Thus, the misspecification of only one of the two models would not cause any harm to the ATE estimator [35].

The proportional hazards assumption for the Cox models had been verified by non-significant deviations from the proportional hazards assumption for each covariate in the model using Schoenfeld residuals.

As statistical programs, we used IBM SPSS Statistics for Windows, Version 24.0 (Armonk, NY: IBM Corp.), Stata Statistical Software, Release 17 (College Station, TX: StataCorp LLC.).

## Results

According to the pre-defined inclusion criteria, the cohort of horizontal switchers included 669 (DMF: 223, TERI: 446 patients) and the cohort of vertical switchers 800 (AZM: 16, CLAD: 27, FTY: 523, NAT: 205, OCR: 21, OZA: 8 patients) RRMS patients, respectively (Table 1). The baseline data of both groups before treatment switch are summarized in Table 2 and show certain imbalances for some baseline variables. Vertical switchers were younger, had a shorter duration of MS and higher values concerning the EDSS, relapse rate and T2-hyperintense and Gd-enhancing T1 lesion loads. Normalized IPW resulted in a weighted sample size of 740 and 698 patients, respectively, and this was well balanced indicated by standardized mean differences (SMD) below 0.1 (Table 2). Mean follow-up was 2.8 years (SD 2.0) for unbalanced horizontal and 3.6 years (SD 2.3) for unbalanced vertical switchers, after IPW 2.7 (SD 1.9) and 3.6 (SD 2.2), respectively.

**Table 1:** Treatment distribution of horizontal and vertical switchers

Treatment	Horizontal switchers *N* = 669	Vertical switchers *N* = 800
DMF	223	0
TERI	446	0
AZM	0	16
CLAD	0	27
FTY	0	523
NAT	0	205
OCR	0	21
OZA	0	8

*AZM* alemtuzumab, *CLAD* cladribine, *DMF* dimethylfumarate, *FTY* fingolimod, *NTZ* natalizumab, *OCR* ocrelizumab, *OZA* ozanimod, *TERI* teriflunomide

**Table 2:** Patient characteristics at timepoint of switch

	Horizontal raw *N* = 669	Vertical raw *N* = 800	SMD raw	Horizontal weighted *N* = 740	Vertical weighted *N* = 698	SMD weighted
Female						
*N*	448	548	− 0.033	499	469	0.003
%	67%	68.5%		67.4%	67.2%	
Age						
Mean	40.2	37.2	0.277	38.3	38.3	0.005
SD	11.3	10.5		11.3	10.5	
Duration of MS (years)						
Mean	8.4	7.6	0.109	7.9	7.8	0.014
SD	7.2	6.5		7.2	6.4	
EDSS						
Mean	1.7	2.2	− 0.356	2.0	2.0	-0.021
SD	1.3	1.4		1.4	1.4	
Relapse rate within 12 months prior treatment switch						
Mean	0.8	1.5	− 0.874	1.2	1.2	0.005
SD	0.8	0.9		1.0	0.8	
≥ 9 T2 lesions						
*N*	554	725	0.232	640	607	0.015
%	82.8%	90.6%		86.5%	87%	
≥ 1 Gd-enhancing T1 lesion						
*N*	190	429	0.530	321	308	0.014
%	28.4%	53.6%		43.4%	44.1%	

*ARR* annualized relapse rate, *EDSS* Expanded Disability Status Scale, *Gd* gadolinium, *MS* multiple sclerosis, *SD* standard deviation, *SMD* Standardized Mean Difference

**Fig. 1:**
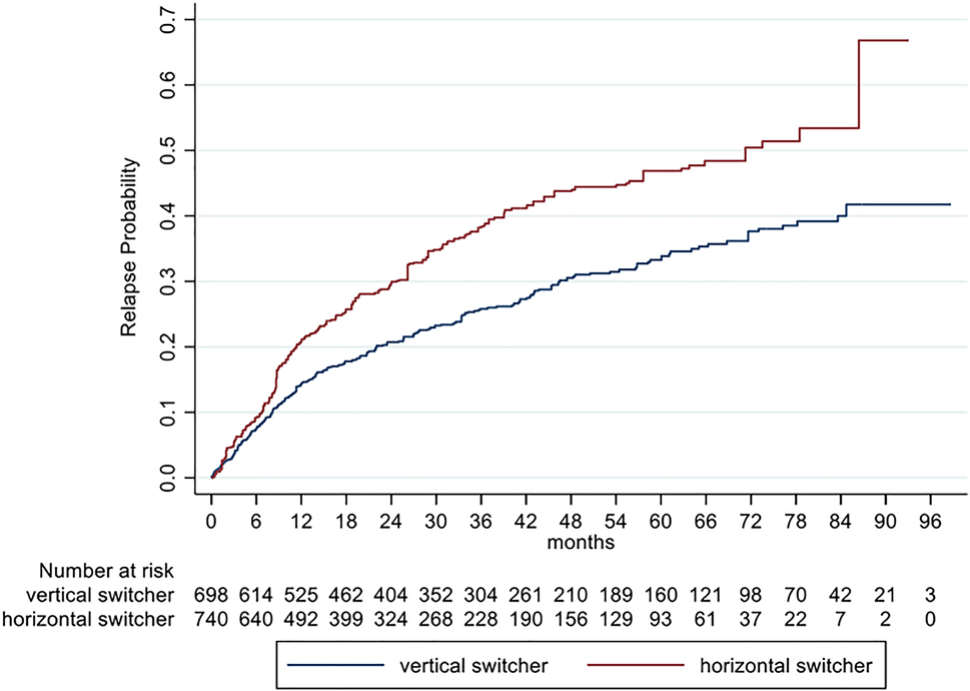
Cumulative probability for experiencing a relapse in RRMS patients comparing horizontal and vertical switchers

Mean annualized relapse rates of the unbalanced cohorts (ARR) were 0.23 (SD 0.61) for horizontal and 0.19 (SD 0.46) for vertical switchers and after IPW 0.39 (SD 0.9) and 0.17 (SD 0.42), respectively. The incidence rate ratio (IRR) in the GLM model for relapses showed an increased relapse probability of 86% for horizontal versus vertical switchers (IRR = 1.86; 95% CI 1.38–2.50; *p* < 0.001). Analyzing the time to the first relapse by Cox regression, a hazard ratio of 1.58 (95% CI 1.24–2.03; *p* < 0.001) indicated an increased risk for relapses of 58% for horizontal switchers (Fig. 1, Table 3).

**Table 3:** Main results in RRMS patients comparing horizontal and vertical switchers

	HR	95% Confidence Interval—lower	95% Confidence Interval—upper	Statistical significance (*p* value)
Cumulative relapse probability	1.58	1.24	2.03	** < 0.001**
Cumulative progression probability sustained for 12 weeks	1.05	0.70	1.57	0.827
Cumulative progression probability sustained for 24 weeks	1.07	0.68	1.67	0.775
Cumulative regression probability sustained for 12 weeks	0.70	0.44	1.10	0.122
Cumulative regression probability sustained for 24 weeks	0.65	0.40	1.07	0.089
Cumulative interrupt probability	1.78	1.46	2.18	** < 0.001**

Bold indicates statistically significant

*HR* Hazard Ratio, *RRMS* relapsing–remitting multiple sclerosis

Mean EDSS change in horizontal switchers was 0.19 (worsening) (95% CI 0.09–0.29) versus 0.14 (worsening) for vertical switchers (95% CI 0.06–0.22), with a difference between treatments of 0.05 (95% CI − 0.08 to 0.17; *p* = 0.451).

**Fig. 2:**
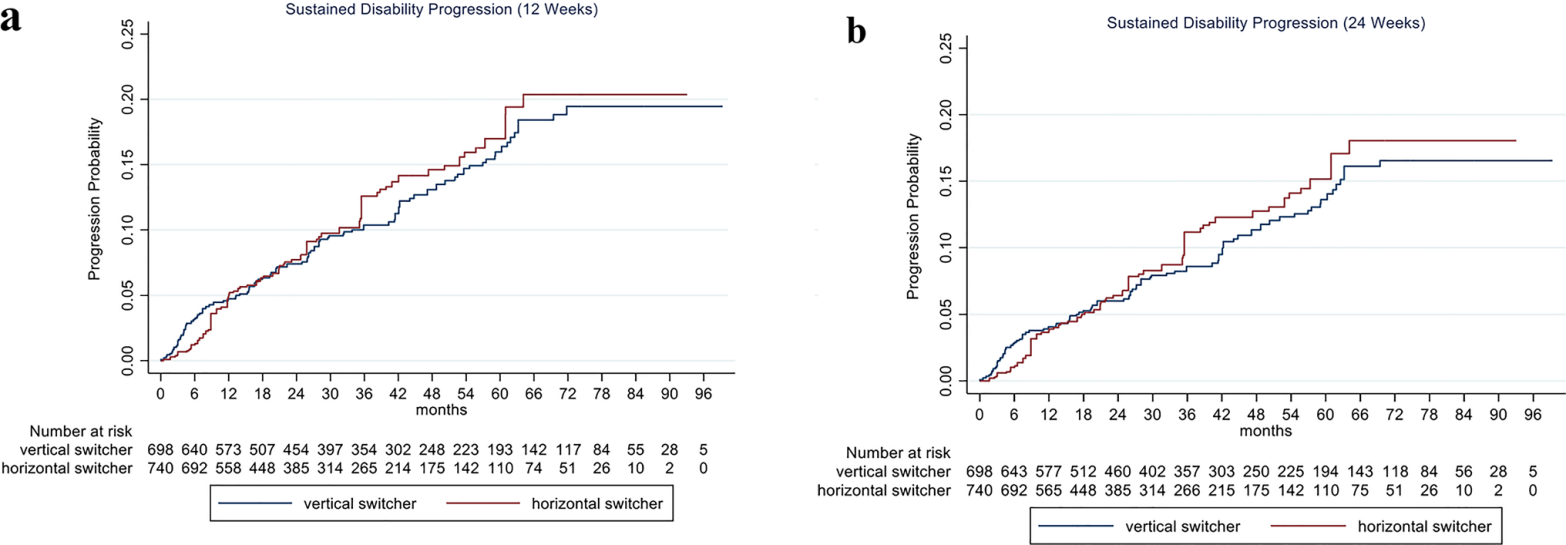
Cumulative probability for disability progression sustained for 12 (**a**) and 24 weeks (**b**) in RRMS patients comparing horizontal and vertical switchers

EDSS progression sustained for 12 weeks and 24 weeks was not significantly different between horizontal and vertical switchers with an increased risk of 5% (HR 1.05, 95% CI 0.70–1.57; *p* = 0.827) and 7% (HR 1.07, 95% CI 0.68–1.67; *p* = 0.775) for horizontal switchers (Fig. 2a and b, Table 3).

**Fig. 3:**
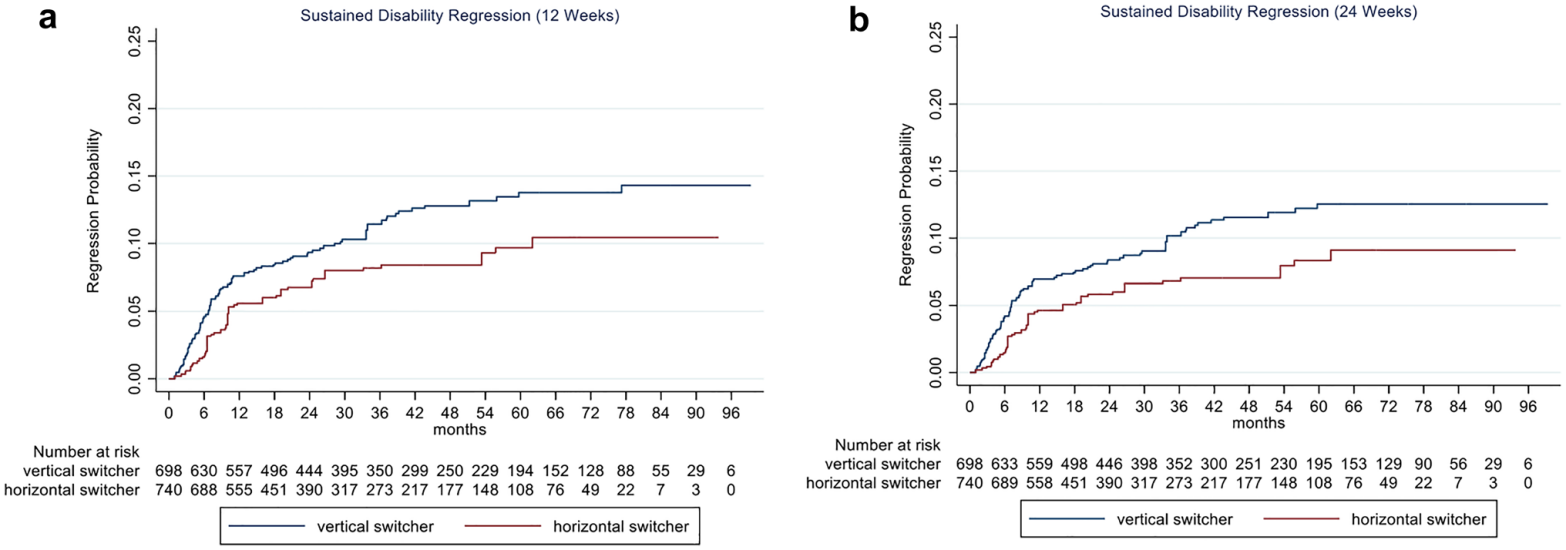
Cumulative probability for disability regression sustained for 12 (**a**) and 24 weeks (**b**) in RRMS patients comparing horizontal and vertical switchers

Sustained EDSS regression for 12 and 24 weeks showed a trend of EDSS improvement in vertical versus horizontal switchers. For horizontal switchers, regression probability was decreased by 30% (HR 0.70, 95% CI 0.44–1.10; *p* = 0.122) and 35% (HR 0.65, 95% CI 0.40–1.07; *p* = 0.089), respectively, compared to vertical switchers (Fig. 3a and b, Table 3).

**Fig. 4:**
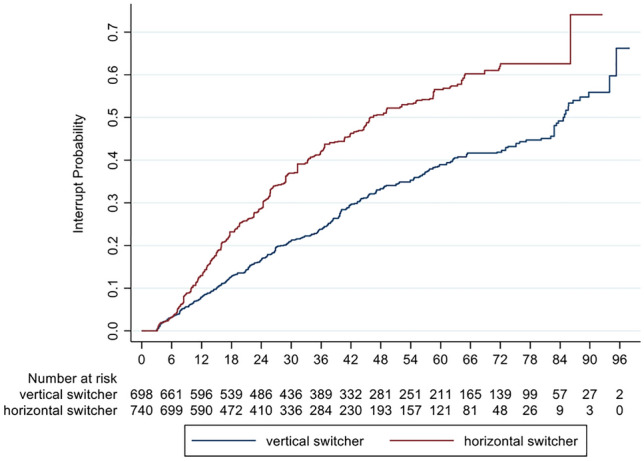
Cumulative probability for treatment interruption in RRMS patients comparing horizontal and vertical switchers

The number of unbalanced patients interrupting treatment was 263 (39.3%) in horizontal switchers (152 stopped and 111 switched to another DMT) and 296 (37%) within vertical switchers (165 stopped and 131 switched to another DMT). The hazard ratio for treatment interruption comparing horizontal versus vertical switch after IPW was 1.78 (95% CI 1.46–2.18; *p* < 0.001) (Fig. 4, Table 3).

Two patients switched from horizontal cohort to AZM, 8 to CLAD, 12 to DMF, 48 to FTY, 14 to NTZ, 6 to OCR, 4 to OZA, 5 to Siponimod (SIPO) and 12 to TERI. In contrast, 10 patients switched from the vertical cohort to AZM, 13 to CLAD, 21 to DMF, 28 to FTY, 31 to NTZ, 19 to OCR, 1 to OZA, 3 to Siponimod (SIPO) and 5 to TERI. Switching patients were only included in the comparative analysis until the timepoint of the treatment switch.

The mean time period until treatment interruption was 26.9 months (SD 18.2) for permanent treatment stop and 21.6 months (SD 16.5) when switching medication within the horizontal cohort and 33.8 months (SD 24) and 28.3 months (SD 19.1) within the vertical cohort, respectively. The mean switching time was 4.2 months (SD: 8.1) for the horizontal and 5.8 months (SD: 10.3) for the vertical cohort.

The reasons for permanent treatment stop within the horizontal group were mainly patients’ wishes (patient’s decision) (*n* = 101), adverse events (AEs) (*n* = 65), disease progression (clinical and/or radiological activity; *n* = 36) and stable disease course (defined at the physician’s and patient’s discretion; *n* = 38), within the vertical cohort the respective reasons were patients’ wishes (*n* = 89), AEs (*n* = 41), disease progression (*n* = 51) and stable disease course (*n* = 37).

The reasons for switching medication within the horizontal group were mainly disease progression (*n* = 93), patients’ wishes (*n* = 66), AEs (*n* = 44), and within the vertical group the reasons were disease progression (*n* = 82), patients’ wishes (*n* = 55) and AEs (*n* = 30). It is important to note that treating neurologists were allowed to name several reasons per patient.

Pregnancy or the wishes to conceive were documented in 11 patients in the horizontal and 25 patients in the vertical cohort.

## Discussion

In this observational study across a nation with well-defined MS treatment units, we analyzed prospectively collected real-world data to compare the effectiveness of change in treatment, frequency and reasons for treatment interruption of DMF and TERI (horizontal switchers) versus AZM, CLAD, FTY, NTZ, OCR and OZA (vertical switchers) after IFN-beta and GLAT therapy in large cohorts of RRMS patients. By nature, the different approved treatment labels caused differences in baseline characteristics of the cohorts (Table 2). To control for these, we used normalized IPW and provided well balanced baseline parameters (Table 2).

There is some discrepancy concerning studies comparing a horizontal and vertical DMT switch [33, 34]. D’Amico et al. analyzed retrospectively 48 RRMS patients with a horizontal switch (IFN-beta and GLAT) and 43 patients with an escalation therapy (FTY, NTZ, cyclophosphamide) after experiencing on-treatment disease activity. No significant differences regarding NEDA 3, in timing to reach the first relapse and obtaining an EDSS of 4.0 were found [33]. In contrast, Chalmer et al. retrieved data from The Danish Multiple Sclerosis Registry on RRMS patients who experienced disease breakthrough on moderately effective therapy (IFN-beta, GLAT, TERI) and switched either to another moderately effective treatment (IFN-beta, GLAT, TERI and DMF) or to a highly effective therapy (FTY and NTZ). Both treatment groups were balanced by propensity score matching and comprised 404 patients. ARR (0.22; IQR 0.19–0.27 versus 0.32; IQR 0.28–0.37) and hazard of reaching a first relapse (HR 0.65; 95% CI 0.53–0.80) were significant lower after a vertical switch. Additionally, no evidence of disability worsening and a weak evidence of disability improvement were found [34].

Comparing our results, we also identified a higher relapse probability and a trend towards less EDSS improvement with a horizontal switch after a platform therapy comparing to a vertical switch. Differences to the aforementioned studies also comprised a treatment switch because of AEs and patients’ wishes. Further, we also included AZM, CLAD, OCR and OZA as a vertical swich therapy. Importantly, in comparison with the aforementioned studies, we analyzed the by far largest study population with a total of 1469 RRMS patients.

Several studies so far demonstrated that patients may stay stable after switching (for various reasons) from IFN-beta and GLAT to DMF and TERI [23–28]. In particular, Buron et al. provided data from the Danish Multiple Sclerosis Registry showing that switching from injectables to DMF and TERI in clinically stable RRMS patients did not increase the risk of disability accumulation [27]. On the contrary, various studies revealed a superior efficacy of highly effective therapies in scenarios where a vertical switch had been performed due to suboptimal response to prior DMTs [29–31]. This contrasts the findings of D’Amico et al. who observed similar efficacy of a lateral switch to IFN-beta compared to an escalation switch to FTY in RRMS patients who failed with other injectables [32].

The hazard ratio for treatment interruption comparing a horizontal versus vertical switch was significantly higher for horizontal switchers. The number of patients interrupting treatment was 263 (39.3%) in the horizontal cohort (152 stopped, and 111 switched) and 296 (37%) within the vertical group (165 stopped and 131 switched). The reasons for permanent treatment interruption within the horizontal group were mainly patients’ wishes and AEs, while within the vertical cohort, respective reasons were patients’ wishes and disease progression. In contrast, the reasons for switching medication within the horizontal and vertical groups were mainly disease progression. These results are in line with various studies evaluating reasons for a switch and treatment discontiuation [18, 21–24]. He et al. also showed a lower hazard of treatment discontinuation (HR 0.55, 95% CI 0.31–0.98) with a vertical (FTY) versus a horizontal (IFN-beta) switch [31]. In our study, almost 80% of patients in the horizontal cohort switched to highly effective therapy, whereas 20% deescalated to a moderately effective treatment within the vertical cohort. In contrast, Patti et al. and Papp et al. documented a lower escalating rate of 53–60% after treatment with DMF and TERI [21, 23]. Furthermore, in 27.3–39.1% of Italian RRMS patients, treatment was deescalated to IFN-beta, GLAT, TERI and DMF after NTZ and FTY therapy in the SWITCH study [21].

The ideal timepoint during a patient’s disease course to start treatment with a DMT or to switch a DMT is difficult to predict. Ziemssen et al. proposed two windows of opportunity for treatment optimization in MS [36]. He postulated that both early initiation of treatment and prompt intervention if disease activity persists despite initial treatment are critical to optimization of treatment outcomes [36]. In line with these notions, several studies provided evidence of superior efficacy of early intensive treatment compared to escalation treatment [37–39].

The major strength of our study is represented by the fact that these analyses are based on a nationwide observational data collection from approved MS facilities, comprising patients in Austria who have been treated with DMF and TERI or AZM, CLAD, FTY, NTZ, OCR and OZA after a treatment with IFN-beta and/or GLAT. The AMSTR represents a secure web-based platform that enables treating neurologists in all Austrian MS centers to immediately perform online documentation during patient visits. To ensure high documentation and data quality in terms of completeness and plausibility, the AMSTR is monitored by an external and independent clinical research organization. Our real-world data show a low ARR, progression rate and discontinuation rate for all drugs reflecting high quality maintenance of MS patients in Austria (data not shown).

On the other hand, an important limitation of our study has to be considered as well when interpreting our results. Information on MRI findings were only available at baseline before start of treatment and we included these as an independent variable for propensity scoring and in the respective outcome models. Information on respective findings during follow-up unfortunately is missing. Furthermore, the reasons for horizontal versus vertical switching could be systematically different in a way not captured by the propensity score, and these cannot be captured by a data-base as used in the AMSTR.

In conclusion, we here present the first large analysis comparing horizontal (DMF and TERI) versus vertical switchers (AZM, CLAD, FTY, NTZ, OCR and OZA) of pwRRMS after a treatment with IFN-beta and/or GLAT. Reasons for switching were manifold including suboptimal treatment response, AEs and convenience. A horizontal switch after a platform therapy resulted in a higher relapse probability and discontinuation rate and a trend towards less EDSS improvement compared to a vertical switch. Therefore, switching to a higher efficacious treatment should be the preferred treatment choice in the case of pwRRMS who do not adequately respond to platform therapies. Given the currently available armamentarium of treatments and considering the findings of our study, a horizontal switch among platform therapies currently shall only be considered in case of side effects of the previous platform therapy.

## Data Availability

The data that support the findings of this study are available on request from the corresponding author.

## References

[1] Gold R, Kappos L, Arnold DL (2012). Placebo-controlled phase 3 study of oral BG-12 for relapsing multiple sclerosis. N Engl J Med.

[2] Fox RJ, Miller DH, Phillips JT (2012). Placebo-controlled phase 3 study of oral BG-12 or glatiramer in multiple sclerosis. N Engl J Med.

[3] O’Connor P, Wolinsky JS, Confavreux C (2011). Randomized trial of oral teriflunomide for relapsing multiple sclerosis. N Engl J Med.

[4] Confavreux C, O'Connor P, Comi G (2014). Oral teriflunomide for patients with relapsing multiple sclerosis (TOWER): a randomised, double-blind, placebo-controlled, phase 3 trial. Lancet Neurol.

[5] Wiendl H, Gold R, Berger T (2021). Multiple Sclerosis Therapy Consensus Group (MSTCG): position statement on disease-modifying therapies for multiple sclerosis (white paper). Ther Adv Neurol Disord.

[6] Kappos L, Radue EW, O’Connor P (2010). A placebo-controlled trial of oral fingolimod in relapsing multiple sclerosis. N Engl J Med.

[7] Calabresi PA, Radue EW, Goodin D (2014). Safety and efficacy of fingolimod in patients with relapsing- remitting multiple sclerosis (FREEDOMS II): a double-blind, randomised, placebo-controlled, phase 3 trial. Lancet Neurol.

[8] Polman CH, O’Connor PW, Havrdova E (2006). A randomized, placebo-controlled trial of natalizumab for relapsing multiple sclerosis. N Engl J Med.

[9] Giovannoni G, Comi G, Cook S (2010). A placebo-controlled trial of oral cladribine for relapsing multiple sclerosis. N Engl J Med.

[10] Giovannoni G, Soelberg Sorensen P (2018). Safety and efficacy of cladribine tablets in patients with relapsing-remitting multiple sclerosis: results from the randomized extension trial of the CLARITY study. Mult Scler.

[11] Cohen JA, Coles AJ, Arnold DL (2012). Alemtuzumab versus interferon beta 1a as first-line treatment for patients with relapsing-remitting multiple sclerosis: a randomised controlled phase 3 trial. Lancet.

[12] Coles AJ, Twyman CL, Arnold DL (2012). Alemtuzumab for patients with relapsing multiple sclerosis after disease-modifying therapy: a randomised controlled phase 3 trial. Lancet.

[13] Hauser SL, Bar-Or A, Comi G (2017). Ocrelizumab versus interferon *β*-1a in relapsing multiple sclerosis. N Engl J Med.

[14] Comi G, Kappos L, Selmaj KW (2019). Safety and efficacy of ozanimod versus interferon *β*-1a in relapsing multiple sclerosis (SUNBEAM): a multicentre, randomised, minimum 12-month, phase 3 trial. Lancet Neurol.

[15] Cohen JA, Comi G, Selmaj KW (2019). Safety and efficacy of ozanimod versus interferon *β*-1a in relapsing multiple sclerosis (RADIANCE): a multicentre, randomised, 24-month, phase 3 trial. Lancet Neurol.

[16] Stuart WH, Cohan S, Richert JR (2004). Selecting a disease-modifying agent as platform therapy in the long-term management of multiple sclerosis. Neurology.

[17] Guger M, Enzinger C, Leutmezer F (2019). Real-life use of oral disease-modifying treatments in Austria. Acta Neurol Scand.

[18] Guger M, Enzinger C, Leutmezer F (2020). Oral therapies for treatment of relapsing-remitting multiple sclerosis in Austria: a 2-year comparison using an inverse probability weighting method. J Neurol.

[19] Fox RJ, Mehta R, Pham T (2022). Real-world disease-modifying therapy pathways from administrative claims data in patients with multiple sclerosis. BMC Neurol.

[20] Bowen J, Mehta R, Pelletier C (2020). Treatment patterns among patients with multiple sclerosis initiating second-line disease-modifying therapy. Adv Ther.

[21] Patti F, Chisari CG, D'Amico E (2020). Clinical and patient determinants of changing therapy in relapsing-remitting multiple sclerosis (SWITCH study). Mult Scler Relat Disord..

[22] Mäurer M, Tiel-Wilck K, Oehm E (2019). Reasons to switch: a noninterventional study evaluating immunotherapy switches in a large German multicentre cohort of patients with relapsing-remitting multiple sclerosis. Ther Adv Neurol Disord.

[23] Papp V, Buron MD, Siersma V (2021). Real-world outcomes for a complete nationwide cohort of more than 3200 teriflunomide-treated multiple sclerosis patients in The Danish Multiple Sclerosis Registry. PLoS ONE.

[24] Bucello S, Annovazzi P, Ragonese P (2021). Real world experience with teriflunomide in multiple sclerosis: the TER-Italy study. J Neurol.

[25] Koch-Henriksen N (2021). It is safe to switch therapy from interferon beta or glatiramer acetate to oral therapy in patients with relapsing multiple sclerosis with stable disease. J Neurol Neurosurg Psychiatry.

[26] Repovic P, Robertson D, Kresa-Reahl K (2021). Effectiveness of dimethyl fumarate in patients with relapsing multiple sclerosis switching after suboptimal response to glatiramer acetate, including patients with early multiple sclerosis: subgroup analysis of RESPOND. Neurol Ther.

[27] Buron MD, Kalincik T, Sellebjerg F (2021). Effect of lateral therapy switches to oral moderate-efficacy drugs in multiple sclerosis: a nationwide cohort study. J Neurol Neurosurg Psychiatry.

[28] Prosperini L, Cortese A, Lucchini M (2020). Exit strategies for "needle fatigue" in multiple sclerosis: a propensity score-matched comparison study. J Neurol.

[29] Vermersch P, Oreja-Guevara C, Siva A (2022). Efficacy and safety of ocrelizumab in patients with relapsing-remitting multiple sclerosis with suboptimal response to prior disease-modifying therapies: a primary analysis from the phase 3b CASTING single-arm, open-label trial. Eur J Neurol.

[30] Weinstock-Guttman B, Bermel R, Cutter G (2022). Ocrelizumab treatment for relapsing-remitting multiple sclerosis after a suboptimal response to previous disease-modifying therapy: a nonrandomized controlled trial. Mult Scler.

[31] He A, Spelman T, Jokubaitis V (2015). Comparison of switch to fingolimod or interferon beta/glatiramer acetate in active multiple sclerosis. JAMA Neurol.

[32] D'Amico E, Patti F, Zanghì A (2018). Lateral switch to IFN beta-1a 44 mcg may be effective as escalation switch to fingolimod in selected persons with relapsing remitting multiple sclerosis: a real-world setting experience. Expert Rev Clin Pharmacol.

[33] D'Amico E, Leone C, Zanghì A (2016). Lateral and escalation therapy in relapsing-remitting multiple sclerosis: a comparative study. J Neurol.

[34] Chalmer TA, Kalincik T, Laursen B (2019). Treatment escalation leads to fewer relapses compared with switching to another moderately effective therapy. J Neurol.

[35] Glynn AN, Quinn KM (2010). an introduction to the augmented inverse propensity weighted estimator. Polit Anal.

[36] Ziemssen T, Derfuss T, de Stefano N (2016). Optimizing treatment success in multiple sclerosis. J Neurol.

[37] Iaffaldano P, Lucisano G, Caputo F (2021). Long-term disability trajectories in relapsing multiple sclerosis patients treated with early intensive or escalation treatment strategies. Ther Adv Neurol Disord.

[38] He A, Merkel B, Brown JWL (2020). Timing of high-efficacy therapy for multiple sclerosis: a retrospective observational cohort study. Lancet Neurol.

[39] Spelman T, Magyari M, Piehl F (2021). Treatment escalation vs immediate initiation of highly effective treatment for patients with relapsing-remitting multiple sclerosis: data from 2 different national strategies. JAMA Neurol.

